# Non-invasive Measurement of Pulmonary Gas Exchange Efficiency: The Oxygen Deficit

**DOI:** 10.3389/fphys.2021.757857

**Published:** 2021-10-21

**Authors:** G. Kim Prisk, John B. West

**Affiliations:** Department of Medicine, University of California, San Diego, La Jolla, CA, United States

**Keywords:** alveolar-arterial PO_2_ difference, A-aDO_2_, pulse oximetry, hypoxemia, Bohr effect

## Abstract

The efficiency of pulmonary gas exchange has long been assessed using the alveolar-arterial difference in PO_2_, the A-aDO_2_, a construct developed by Richard Riley ~70years ago. However, this measurement is invasive (requiring an arterial blood sample), time consuming, expensive, uncomfortable for the patients, and as such not ideal for serial measurements. Recent advances in the technology now provide for portable and rapidly responding measurement of the PO_2_ and PCO_2_ in expired gas, which combined with the well-established measurement of arterial oxygen saturation *via* pulse oximetry (SpO_2_) make practical a non-invasive surrogate measurement of the A-aDO_2_, the oxygen deficit. The oxygen deficit is the difference between the end-tidal PO_2_ and the calculated arterial PO_2_ derived from the SpO_2_ and taking into account the PCO_2_, also measured from end-tidal gas. The oxygen deficit shares the underlying basis of the measurement of gas exchange efficiency that the A-aDO_2_ uses, and thus the two measurements are well-correlated (*r*^2^~0.72). Studies have shown that the new approach is sensitive and can detect the age-related decline in gas exchange efficiency associated with healthy aging. In patients with lung disease the oxygen deficit is greatly elevated compared to normal subjects. The portable and non-invasive nature of the approach suggests potential uses in first responders, in military applications, and in underserved areas. Further, the completely non-invasive and rapid nature of the measurement makes it ideally suited to serial measurements of acutely ill patients including those with COVID-19, allowing patients to be closely monitored if required.

## Introduction

For the lung to exchange gas (O_2_ from the inspired air into the blood, and CO_2_ from the blood to the expired gas), alveolar gas and pulmonary capillary blood must be brought into close apposition across the thin alveolar-capillary membrane. Any degree of spatial mismatch between ventilation and perfusion [ventilation-perfusion (VA/Q) inequality] will lower the efficiency of the exchange of any gas, resulting in a difference between the partial pressure of a gas in the arterial blood leaving the lung, and gas in the exhaled breath (Rahn and Fenn, 1955; West, 1969).

For O_2_ and CO_2_, the dissociation curves that describe the content of the gas in blood as a function of partial pressure, are markedly different. The sigmoidal shaped O_2_ dissociation curve rapidly flattens at higher values of PO_2_. Thus, the presence of any regions of the lung with a low VA/Q ratio will result in the addition of poorly oxygenated blood to the arterial circulation, but a compensatory increase in overall ventilation (from chemoreceptive responses) cannot add more oxygen to blood exiting regions of high VA/Q. In contrast, the quasi-linear CO_2_ dissociation curve means that low and high VA/Q regions can compensate for each other. Thus, it is common to see patients with pulmonary disease with arterial hypoxemia, while having a normal arterial PCO_2_ (West and Luks, 2016).

A small increase in VA/Q inequality occurs with healthy aging (Cardus et al., 1997), and increased VA/Q inequality is a hallmark of virtually all pulmonary diseases (Hopkins and Wagner, 2017). Therefore, the measurement of the alveolar-arterial PO_2_ difference (A-aDO_2_) has long been a mainstay in assessing the disruption to pulmonary gas exchange caused by disease (Filley et al., 1954).

## Historical Context Of Measuring the Alveolar-Arterial Difference in Po_2_

While conceptually simple, measuring the A-aDO_2_ is technically challenging. Richard Riley first showed that the PO_2_ in arterial blood could be measured by equilibrating a small bubble of air with the blood and measuring the PO_2_ in the gas (Riley et al., 1957). However, at the time, the technical difficulties of reliably sampling alveolar gas were overwhelming. To bypass this problem, Riley developed the construct of the “ideal alveolar PO_2_.” This was the alveolar PO_2_ in the lung that *would have been present* if there was no ventilation-perfusion inequality, the PCO_2_ in the alveolar gas was the same as that in arterial blood, and the respiratory exchange ratio was the same as that in the actual lung.

The ideal alveolar PO_2_ can be obtained from an arterial blood sample using the alveolar gas equation (Rahn and Fenn, 1955):

$$\mathrm{ideal}\;{\mathrm{PAO}}_{2}={\mathrm{PIO}}_{2}-{\mathrm{PaCO}}_{2}/R-\left[{\mathrm{PaCO}}_{2}*{\mathrm{FIO}}_{2}*\left(1-R\right)/R\right]$$

where A refers to alveolar, a to arterial, I to inspired, and R is the respiratory exchange ratio, the CO_2_ production divided by the O_2_ consumption (generally assumed to be 0.8 at rest). The final term in this equation is often ignored as it typically has a magnitude of only a few mmHg. A more detailed description of Riley’s innovative approach can be found in West et al. (2020). This approach provides a number representing the alveolar PO_2_, and it does so without actually measuring alveolar gas.

## The Alternative Approach of the Oxygen Deficit

Compact, rapidly responding gas analysis devices are now readily available, allowing direct measurement of expired PO_2_ and PCO_2_. The Riley construct utilizes the ideal alveolar PO_2_ to obviate the need to make a “technically difficult” measurement to calculate the A-aDO_2_. The oxygen deficit (OD) comes from a direct measurement of expired gas partial pressures and uses a non-invasive means to determine what would otherwise be an invasive measurement, the arterial PO_2_.

**Figure 1:**
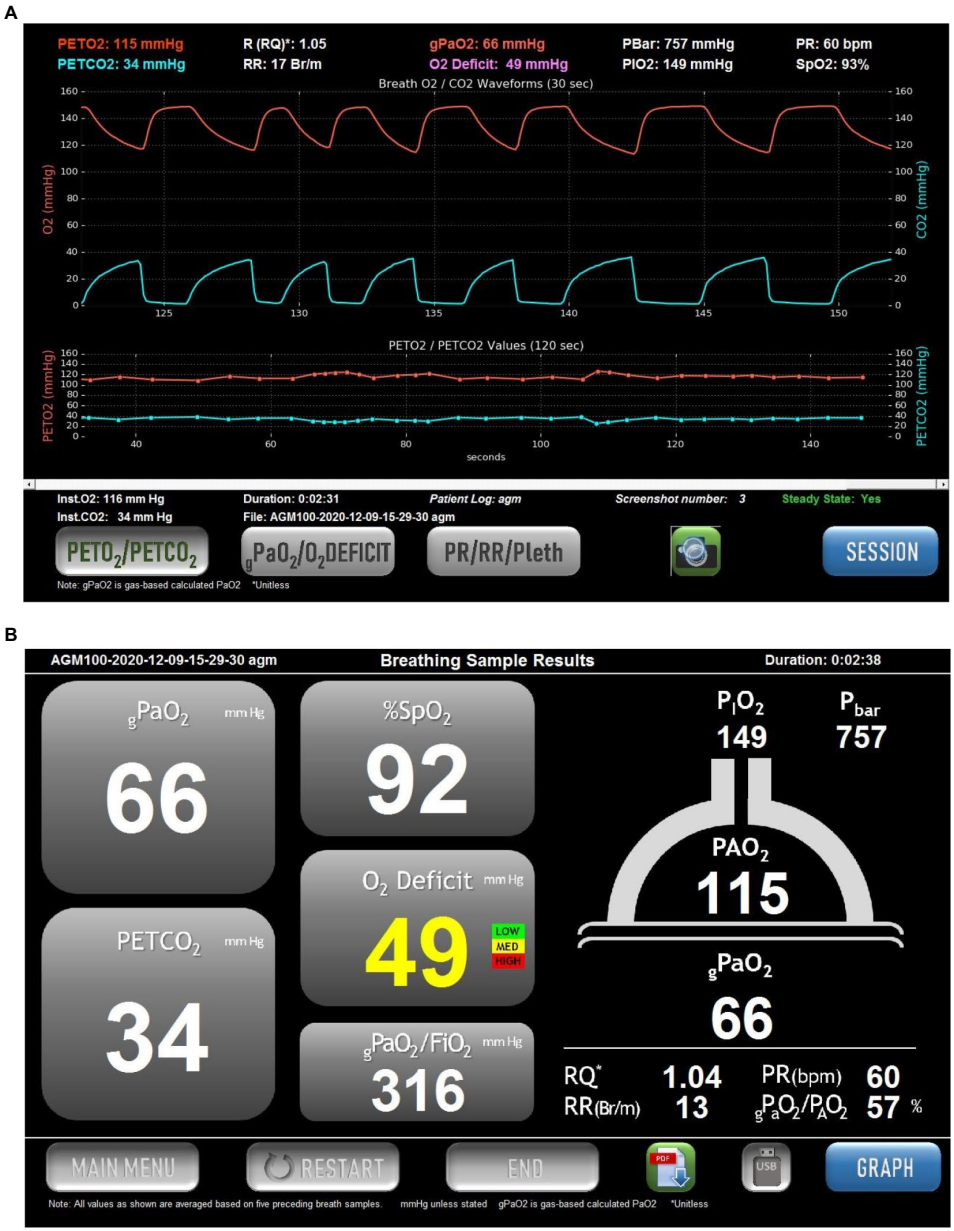
Example of the screens displayed in the commercial version of the Alveolar Gas Meter (AGM100^TM^, MediPines Corp, Costa Mesa, CA). Data are taken from a spontaneously breathing patient suffering from COVID-19. **(A)** Continuous records of PO_2_ (red) and PCO_2_ (blue) over a 30-s period of quiet breathing (upper traces). Note that in this patient there is a steep alveolar plateau for O_2_ and CO_2_, indicative of marked heterogeneity in the lung. Below these are plots of end-tidal PO_2_ (red) and PCO_2_ (blue) over the preceding 150s. The lower traces allow an assessment of whether the patient is in a steady state. This is also indicated by the Steady State indicator in the lower right of the screen. At the top of the screen are numerical values for the end-tidal partial presses of O_2_ and CO_2_, the respiratory quotient (RQ), respiratory rate (RR), barometric pressure (PBar), inspired PO_2_ (PIO_2_), pulse rate (PR) and arterial oxygen saturation *via* pulse oximetry (SpO_2_). In the center at the top is the calculated arterial PO_2_ (termed gPaO_2_, red text), and the O_2_ Deficit (the difference between end-tidal PO_2_ and calculated arterial PO_2_). **(B)** A screen summarizing the data from **(A)** without the graphical displays. The cartoon of the lung on the right shows the measured end-tidal PO_2_ (115mmHg in this example), and the calculated arterial PO_2_ (gPaO_2_, 66mmHg in this example), which together result in the oxygen deficit in traffic-light color-coded text (49mmHg in this example). The operator may toggle between this screen and that in **(A)** as desired.

The approach is to continuously measure expired O_2_ and CO_2_ while the patient breathes quietly on a mouthpiece while wearing a noseclip. The final concentrations measured just before the abrupt transition to inspired gas are taken as the end-tidal values of PO_2_ and PCO_2_. An example of the expired gas record is shown in Figure 1. The end-tidal values for partial pressure are a good reflection of the values within the alveolus (discussed in detail in reference West et al., 2020) and are highly reproducible. Previous work has shown that the breath-to-breath within-subject standard deviation of normal subjects breathing air is ~1.4mmHg for PO_2_ and ~0.7mmHg for PCO_2_ (West et al., 2020), with somewhat lower numbers when breathing a hypoxic gas. A trend plot of the last 30 values of these (covering 1–2min) provides a direct indication of whether the patient is in steady-state, an important consideration since highly variable breathing would result in considerable variation in end-tidal partial pressures for both O_2_ and CO_2_.

Having directly measured the alveolar partial pressure for O_2_ (and CO_2_), the A-aDO_2_ could be measured directly by taking an arterial blood sample to measure arterial PO_2_. However, to make the process both rapid and non-invasive, arterial PO_2_ is estimated from the arterial oxygen saturation (SaO_2_) as measured by pulse oximetry (SpO_2_) as determined simultaneously using a fingertip pulse oximeter. This is then used to determine the corresponding arterial PO_2_ from the Hill equation:

$${\mathrm{PO}}_{2}{}^{n}=P_{50}{}^{n}*{\mathrm{SaO}}_{2}/\left(1-{\mathrm{SaO}}_{2}\right)$$

where P_50_ is the PO_2_ at a saturation of 50%, and the SaO_2_ is expressed as the fractional saturation; range [0, 1]. PO_2_ is determined by taking the logarithm of the equation and solving algebraically.

The term n (commonly referred to as the “Hill-n”) is that required to match the sigmoidal shape of the O_2_-Hb dissociation curve, and a value of 2.7 is commonly used (Severinghaus, 2002; Prisk and West, 2019). While a Hill-n of 2.7 provides an excellent fit to the experimentally determined values of saturation and PO_2_ over the entire range of saturation (Severinghaus, 1966; Prisk and West, 2019), in practice, only blood oxygen saturations in the range of 75–100% are likely to be encountered in patients. Over this limited range, an improved fit to the experimental data is achieved with a Hill-n=2.88 (Liu et al., 2020).

The P_50_ of blood is normally ~27mmHg, however this varies with alterations in PCO_2_, body temperature, base excess, and levels of 2,3-diphosphoglycerate (2,3-dpg; West and Luks, 2016). Because the end-tidal gas partial pressure reflects the alveolar PCO_2_, the leftward or rightward shift of the O_2_-Hb dissociation curve from a PCO_2_ different to the normal value of 40mmHg (the Bohr effect) can be accounted for. Using the Kelman routines (Kelman, 1966, 1968) an empiric relationship for P_50_ assuming otherwise normal conditions for temperature, base excess and 2,3-dpg is determined (Prisk and West, 2019). This is used to correct for changes in alveolar PCO_2_, assuming this is equal to arterial PCO_2_, an equivalence that has been long established (Comroe and Dripps, 1944). Alterations in base excess, 2,3-dpg, or body temperature are not accounted for, because a blood sample is not obtained.

The difference between the calculated arterial PO_2_ and the measured end-tidal (alveolar) PO_2_ is termed the OD. This can be thought of as a surrogate measurement of the A-aDO_2_. In the latter case the arterial value is measured, and the alveolar value estimated as described by Riley (above), while in the case of the OD, the alveolar value is measured, and the arterial value estimated. This should not be confused with the “oxygen deficit” that provides a measure of the anaerobic contribution during exercise (Krogh and Lindhard, 1920; Medbo et al., 1988).

## Limitations of the Measurement of the Oxygen Deficit

In the normal lung there is variation in the regional alveolar PO_2_, and this is often exaggerated in lung disease. The expired gas is a mixture of gas from all regions of the lung, just as the arterial blood is a mixture of blood from all regions of the lung. Further as gas exchange continues throughout expiration, PO_2_ continues to fall. However, provided the end-tidal values are measured at functional residual capacity (FRC), the naturally occurring volume for end expiration at rest, the effect of ongoing gas exchange is minimal. Thus, the end tidal PO_2_ is a direct and useful measurement of the alveolar PO_2_ (West et al., 2020).

The obvious challenge of determining the OD is the estimation of the arterial PO_2_ from the SpO_2_ given the shape of the O_2_-Hb dissociation curve which is very flat at higher values of PO_2_. At high values, even small errors in SpO_2_ translate into large differences in the calculated PaO_2_. However, this problem becomes smaller at lower values of SpO_2_ as the O_2_-Hb dissociation curve becomes steeper. A study addressing the likely errors in calculated PaO_2_ showed that for SpO_2_ values of 94% and below, the error in the calculated PaO_2_ was less than 5mmHg (Prisk and West, 2019). Above a SpO_2_ of 94% the calculation of PaO_2_ was, as expected, unreliable. However, if SpO_2_ is greater than 94% while breathing air at sea level, then no major gas exchange impairment exists, and so there is no need to measure OD.

Because the approach considers the alveolar PCO_2_ as well as the PO_2_, the left or right shift in the O_2_-Hb dissociation curve from changes in alveolar PCO_2_ (the Bohr effect) can be directly accounted for. This effect is the principal cause of shifts in the O_2_-Hb dissociation curve, and so the ability to correct for this is critical. Failure to do so would result in errors in the OD of >5mmHg for SpO_2_ values of 94% (see figure 4 of Prisk and West, 2019 for details). Shifts in the O_2_-Hb dissociation curve from other causes (base excess, temperature, 2–3 dpg) are not taken into account with the non-invasive approach. These however, are much smaller, and produce only minor errors in the calculated OD (Prisk and West, 2019). A Monte Carlo simulation of the typical simultaneously present errors in the measurements of SpO_2_ and alveolar PCO_2_ showed that the calculated OD had a slight negative bias (<5mmHg) and typical variability of ~5mmHg at an SpO_2_ of 94%, with both of these values decreasing as SpO_2_ fell, showing the viability of the approach (Prisk and West, 2019).

## Oxygen Deficit in Normal Subjects

Initial studies in normal subjects were performed with the subjects breathing a hypoxic gas mixture (FIO_2_=0.125) to ensure that the SpO_2_ fell into the range in which OD could be reliably measured (SpO_2_<95%). A study of 20 young subjects (19–31years) and 11 older subjects (47–88years) showed a very small OD in the young cohort (~2mmHg), which was increased in the older (~8mmHg; West et al., 2018b). The increase in OD with increasing age is consistent with the well-known increase in the A-aDO_2_ with healthy aging (Raine and Bishop, 1963).

A more extensive subsequent study explored the effects of varying the inspired oxygen between the previously used hypoxic gas (FIO_2_=0.125) up to and including breathing air (FIO_2_ values of 0.15, 0.175, and 0.21; Liu et al., 2020). This study again showed a higher OD in the older cohort compared to the young, with the difference persisting at all values of inspired oxygen, including air. Importantly, there was no statistical difference in the measured values of OD between and FIO_2_ of 0.125 and 0.175, although OD rose in both cohorts when the subjects were breathing air. The result is consistent with an expected reduction in the A-aDO_2_ as inspired PO_2_ is lowered due to minimization of the effect of VA/Q inequality as the saturation falls and gas exchange occurs on the steeper and more linear portion of the O_2_-Hb dissociation curve. The intra-subject variability in the measured OD was large at high values of SpO_2_, and fell rapidly as SpO_2_ was reduced below 94%, consistent with the simulation studies performed (Prisk and West, 2019). The study showed that the OD was sensitive to the mild gas exchange impairment associated with healthy aging, even while breathing air, but that individual errors at high values of SpO_2_ meant that the measurement was not likely to be useful in individual subjects at SpO_2_ values above 94%.

A recent study has also demonstrated the validity of the non-invasive approach to measure a gas exchange deficit. A direct comparison between OD and arterial blood gas (ABG) was performed in 25 normal subjects during hypoxic exercise, showing a correlation between OD and A-aDO_2_ with an *r*^2^=0.71, and with a small bias between the two, with OD being on average 5.2±5.0mmHg higher than A-aDO_2_ (Howe et al., 2020). The non-invasive nature of the measurement serves to enable measurements in conditions in where it would be challenging to perform ABGs, and in particular, serial ABGs. Recent examples are measurements performed in trained breath-hold divers before and after dives at an open-water dive site (Patrician et al., 2021a,b). The studies showed a substantial but transient decrement in gas exchange efficiency as measured by increased OD, in some cases to nearly 70mmHg. This was likely due to the development of pulmonary edema from the hydrostatically induced lung compression (lung-squeeze).

## Oxygen Deficit in Patients with Lung Disease

A small initial study in a cohort of ambulant patients from a general pulmonary outpatient clinic with a variety of pulmonary diseases showed that the OD was greatly elevated in this group compared to normal, with an average OD of ~49mmHg (West et al., 2018a). When the OD was directly compared to the A-aDO_2_ measured by the collection of an ABG in 23 patients with an SpO_2_ of 95% or less, there was a high correlation (*r*^2^=0.72; West et al., 2018c). There were similar strength correlations between the calculated PaO_2_ and that measured from the ABG, and between end-tidal PCO_2_ and that from the ABG. The calculated PaO_2_ was on average ~4mmHg higher than that measured from the ABG. This study showed that the non-invasive approach provided a convenient, low cost, and accurate alternative to the use of an ABG to measure the magnitude of the gas exchange disruption in patients with pulmonary disease.

A recent case report highlighted the use of the non-invasive approach in determining the underlying cause of a gas exchange defects in a patient in whom ABGs could not readily be obtained (Amaza et al., 2021). This report served to highlight the potential of the non-invasive approach, and further showed that the approach was useful as a teaching tool.

In the context of the ongoing SARS-CoV-2 pandemic, a small preliminary study investigated the usefulness of the non-invasive approach to measuring the impairment of pulmonary gas exchange in patients with suspected COVID-19 considered to be at risk of deterioration before obvious respiratory failure had ensued (McGuire et al., 2021). Patients were either breathing air, or on supplemental low-dose oxygen, which was temporarily discontinued for ~10min before measurements were taken. Of 13 patients studied, five were discharged home and the other eight were admitted based on physician decision using standard procedures. The OD was significantly greater in the patients who were admitted than in those who were discharged (OD 55±20 vs. 32±14 respectively, *p*=0.041), suggesting that the measurement was a potentially useful means of assessing severity. Similarly, the oxygen deficit was significantly higher in the patients requiring supplemental O_2_ than in those who did not (65±9 vs. 30±1 respectively, *p*<0.001) again suggesting that the non-invasive measurement of gas exchange impairment provided useful clinical insight in a rapid non-invasive manner.

## Discussion

**Figure 2:**
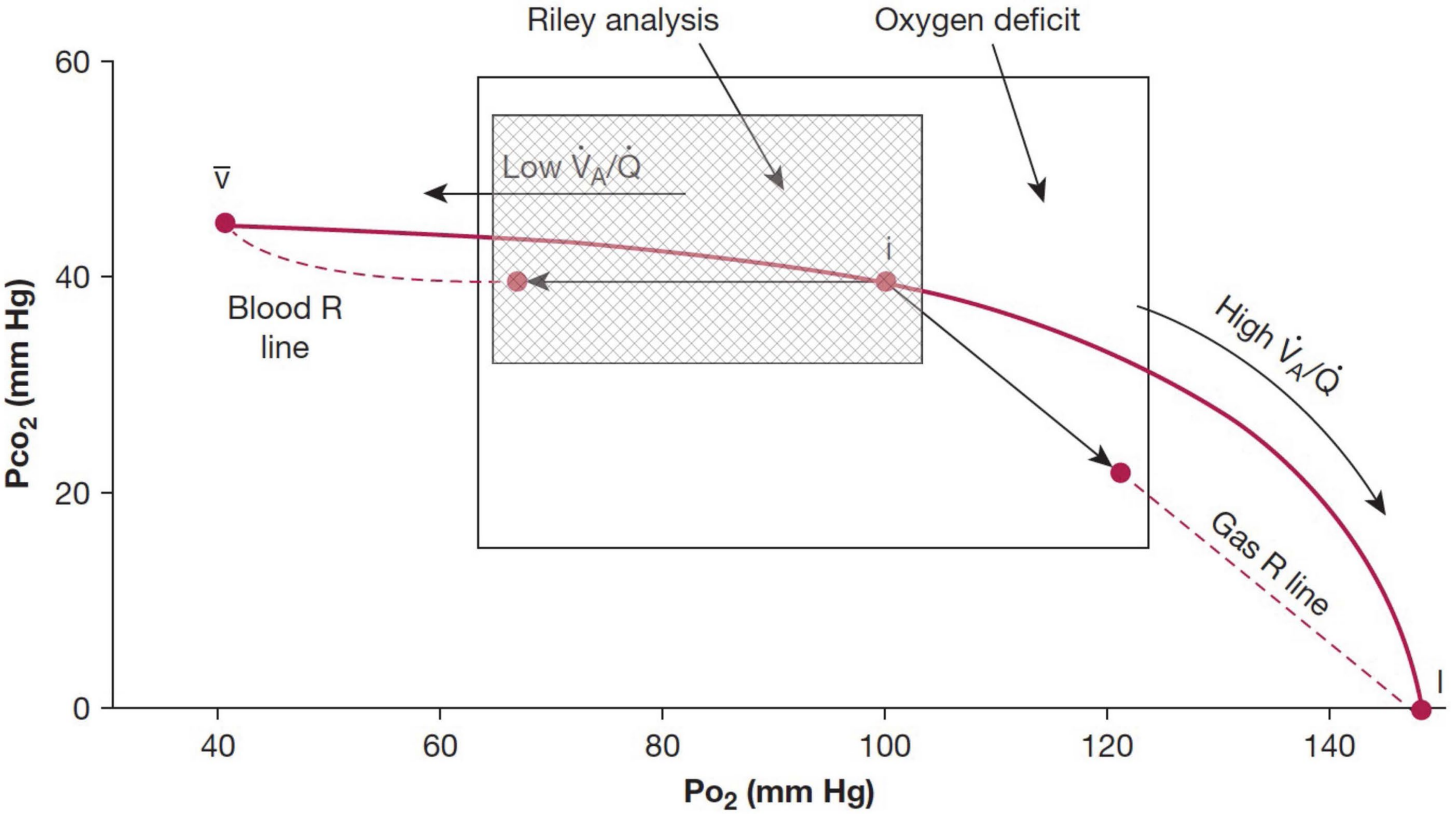
Classic O_2_-CO_2_ diagram with the ventilation-perfusion line joining the points for mixed venous blood and inspired gas. The traditional Riley analysis is based on the composition of arterial blood and ideal alveolar gas, and it is strongly biased by lung units with low ventilation-perfusion ratios that lie to the left of the ideal point (hatched area). By contrast, the new test also includes contributions from lung units with high ventilation-perfusion ratios that are located to the right of the ideal point. Modified from West et al. (2018a).

The studies performed to date show that a non-invasive approach can be used to quantitatively assess the gas exchange deficit in patients with pulmonary disease. The approach taken is in many respects comparable to the traditional measurement of the A-aDO_2_ first developed by Riley, and there is considerable physiological overlap in the measurements. This is shown in Figure 2 which shows the classic O_2_-CO_2_ diagram and highlights the aspects of the VA/Q inequality that the two methods encompass.

The traditional method of Riley focuses on the consequences of the presence of regions of low VA/Q that serve to add end-capillary blood with a low PO_2_ to the arterial blood (so-called venous admixture), the result being arterial hypoxemia and an increased A-aDO_2_. The measurement of oxygen deficit encompasses the effects of low VA/Q and high VA/Q, although the effect of high VA/Q regions on arterial PO_2_ is small because of the shape of the O_2_-Hb dissociation curve. The large overlap in the areas of influence of the two methods (Figure 2) means that the two measurements would be expected to be highly correlated, albeit not the same, and this was demonstrated with a strong correlation between measures of A-aDO_2_ and OD (West et al., 2018c; Howe et al., 2020).

It is reasonable to question what additional information is gained by measuring the OD as opposed to simply measuring SpO_2_. While both VA/Q mismatch and shunt will serve to decrease arterial PO_2_ (and thus SpO_2_) and increase OD, so too will hypoventilation. Because the alveolar PCO_2_ is also measured, hypoventilation can readily be detected which may provide an important clinical distinction of the cause of hypoxemia in some patients. Further, the OD takes into account the effect of changes in PCO_2_ on the O_2_-Hb dissociation curve. Thus, the oxygen deficit directly addresses the efficiency of gas exchange, in the same way that the A-aDO_2_ does.

The important clinical measurement of the A-aDO_2_ has been performed using an invasive approach for ~70years. However, its use has become less common in recent years, likely due to the cost, time required for the measurement, and the uncomfortable and invasive nature of the procedure. In contrast, the OD is a rapid, non-invasive measurement that can be readily performed on patients ranging from ambulatory to those on mechanical ventilation. The measurement takes only a few minutes, requiring only that the patient breathe quietly on a mouthpiece while wearing a noseclip for ~2min, while wearing a fingertip pulse oximeter. The equipment is portable, making it suitable for use not only in the hospital, but in the field, and in underserved areas.

## Author Contributions

GP wrote, edited, and approved the article. JW edited and approved the article. All authors contributed to the article and approved the submitted version.

## Funding

Support for some was provided by MediPines Corporation which provided AGM100TM units for use in some of the studies referenced. Support was provided by MediPines Corporation to cover publication costs.

## Conflict of Interest

The University of California, San Diego, has exclusively licensed technology to MediPines Corporation, Orange County, CA, to develop a device (the AGM100TM) used in some studies referenced in this work. JW declares a financial interest with MediPines Corporation.

The remaining author declares that the research was conducted in the absence of any commercial or financial relationships that could be construed as a potential conflict of interest.

## Publisher’s Note

All claims expressed in this article are solely those of the authors and do not necessarily represent those of their affiliated organizations, or those of the publisher, the editors and the reviewers. Any product that may be evaluated in this article, or claim that may be made by its manufacturer, is not guaranteed or endorsed by the publisher.
